# Supplementary material for: The MEK-ERK-MST1 Axis Potentiates the Activation of the Extrinsic Apoptotic Pathway during GDC-0941 Treatment in Jurkat T Cells
Source: Cells. 2019 Feb 21;8(2):191. doi: 10.3390/cells8020191 (PMC6406719; doi:10.3390/cells8020191)
Supplement: Supplementary file 1 [file cells-08-00191-s001.pdf]

Protein	Peptide Sequence	Control (Total Area)	GDC-0941 (Total Area)	GDC/CTRL Ratio
NEK2	SDGGHTVLHR	23996	1948381	81
NEK2	ILNHDTSFAK	286600	28686594	101
NEK2	YSDELNEIITR	902326	75235560	83
PLK1	AGVPGVAAPGAPAAAPPAK	2133964	51946708	24
PLK1	SLLLKPHQR	779849	18754126	24
PLK1	LILYNDGDSLQYIER	1833385	57238208	31

Figure 1S. Quantification of NEK2- and PLK1-specific peptides co-precipitated with MST1 protein in control and GDC-0941 treated cells. Individual peptides were identified and quantified in trypsin-digested mixture of proteins which were co-precipitated with MST1 protein using LC-MS/MS analysis.
